# Kikuchi-Fujimoto Disease in a Young Female: A Case Report and Literature Review

**DOI:** 10.7759/cureus.19321

**Published:** 2021-11-06

**Authors:** Aadil M Khan, Moinuddin Ahmad, Owaise Muhammad, Shafaq Taj, Saher T Shiza

**Affiliations:** 1 Internal Medicine, Ganesh Shankar Vidyarthi Memorial Medical College, Kanpur, IND; 2 General Medicine, Lugansk State Medical University, Kyiv, UKR; 3 Internal Medicine, Deccan College of Medical Sciences, Hyderabad, IND

**Keywords:** lymphoma, fever, immunohistochemistry, lymphadenopathy, kikuchi-fujimoto disease

## Abstract

Kikuchi-Fujimoto disease (KFD) is histiocytic necrotizing lymphadenitis, a rare immune-mediated disorder presenting with lymphadenopathy, leukopenia, and occasionally fever. Herein we report a case of KFD who presented with anorexia, fever, and cervical lymphadenopathy. Lymph node biopsy and immunohistochemistry confirmed the diagnosis of KFD. She was treated with prednisolone and paracetamol, and her condition improved gradually on subsequent follow-up. A patient presenting with fever and lymphadenopathy leads to prompt investigations for common diseases such as tuberculosis and lymphoma. However, rare diseases like KFD must be kept in mind, and a lymph node biopsy followed by histopathologic examination and immunohistochemistry should be done to confirm the diagnosis.

## Introduction

Kikuchi-Fujimoto disease (KFD) is histiocytic necrotizing lymphadenitis, a rare self-limiting disease that is relatively benign in its course and often manifests with fever and focal lymphadenopathy. Although this disease is commonly diagnosed in Asian women, it equally affects both sexes [1]. It is a disease of the young, especially those aged 30 years or less, including children, and presents with fever, weight loss, focal lymphadenopathy, generalized lymphadenopathy, splenomegaly, and hepatomegaly [1-3]. Given the overlapping signs and symptoms of presentation, it is not unusual to mistake KFD for lymphoma, lymphoproliferative disorders, and other autoimmune disorders [4]. A viral-induced etiology has also been considered in disease pathogenesis, even though exact etiology remains unclear [5]. Symptomatic treatment has been used in the management as KFD tends to self-resolve within few days to weeks even without medication, with rare exceptions to steroids which have been indicated only for severe and refractory cases [4,5].

## Case presentation

A 22-year-old girl presented with complaints of fever, fatigue, anorexia, and cervical lymphadenopathy for the last two months. The fever was intermittent and mild, with no rigors and chills. Fatigue was present most of the day. She did not report any change in the size of lymph nodes over the last two months and denied sore throat, chronic cough, night sweats, otalgia, dysphonia, dysphagia, rash, or weight loss. She had no history of any chronic disease, malignancy, illicit drug use, and alcohol abuse. She took over-the-counter antipyretics and antibiotics without any resolution of symptoms and neck mass.

On examination, she looked pale, anxious, well oriented in time, place, and person. She had a temperature of 100^o^F and respiratory rate of 19/minute, blood pressure of 110/70 mmHg, heart rate of 85/minute, and oxygen saturation of 97%. There were firm, non-tender, palpable cervical lymph nodes bilaterally, with a maximum size of 2 cm x 3 cm. There was no evidence of erythema, warmth, or edema. Oral and otolaryngic examination was normal. Her respiratory and cardiovascular examination was unremarkable, with vesicular breathing and normal heart sounds. Her abdomen was soft, non-tender with no organomegaly.

The results of the ordered blood investigations are shown in Table 1. Her kidney and liver functions were unremarkable with no hematuria, pyuria, or jaundice. Her chest x-ray was normal. Viral screening for hepatitis B, C, and human immunodeficiency virus was negative. Echocardiogram and abdominopelvic ultrasound were within normal limits. Neck ultrasonography confirmed cervical lymphadenopathy with preserved lymph node architecture. The autoimmune screening was performed, and the results are shown in Table 2.

**Table 1:** Results of initial blood workup. Hb: hemoglobin, TLC: total leukocyte count, RBC: red blood cells, ESR: erythrocyte sedimentation rate, CRP: c-reactive protein, LDH: lactate dehydrogenase.

Parameter	Patient value	Normal range
Hb	11.7 gm/dL	11.0-15.0
TLC	12000 cells/mm^3^	4000-11000
Neutrophils	79%	40-75
Lymphocytes	15%	20-50
Eosinophils	3%	1-6
Monocytes	2%	2-10
Basophils	1%	<1
Platelet count	250,000 cells/mm^3^	150,000-350,000
RBC count	4.1 x 10^6^ cells/mm^3^	4-6
ESR	51	<15
CRP	3.2 mg/dL	<0.3
LDH	376 U/L	230-460

**Table 2:** Results of autoimmune screening. ANA IFA: antinuclear antibody immunofluorescence assay, Anti-dsDNA: anti-double-stranded deoxyribonucleic acid.

Antibody	Status
ANA IFA	Positive
Pattern	Nuclear fine speckled
Intensity	1+
Primary titer/dilution	1:100
Endpoint titer/dilution	1:100
Anti-dsDNA	17 IU/ml

**Figure 1:**
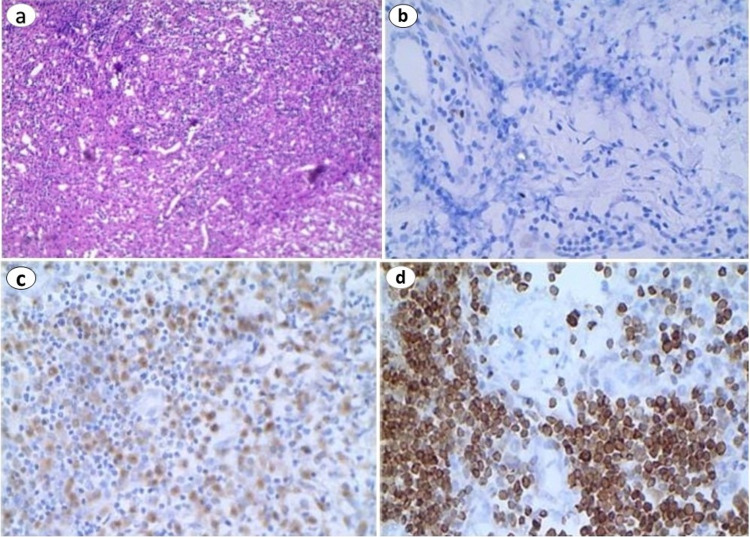
Hematoxylin-eosin staining of cervical lymph node demonstrating lymph node hyperplasia and histiocytosis (a). Immunohistochemical staining of cervical lymph node demonstrating Ki67 (b), CD68 (c), and BCL2 protein (d).

A provisional diagnosis of lymphoma was made based on history, clinical examination, blood investigations, and imaging study. An excisional cervical lymph node biopsy and immunohistochemistry were performed. Biopsy revealed partially affected architecture and areas of necrosis, nuclear dust, and mononuclear cells within surrounding tissues (Figure 1a). Immunohistochemistry of lymph nodes was immunoreactive for CD68 (Figure 1c), Ki67 (Figure 1b), and positive for BCL2 protein (Figure 1d), consistent with a diagnosis of KFD.

She was managed with oral prednisolone 30 mg daily and paracetamol. Her fever and fatigue subsided gradually after starting prednisolone. On follow-up after one month, she was asymptomatic with a marked reduction in the erythrocyte sedimentation rate (ESR) and the size of cervical lymph nodes.

## Discussion

Kikuchi and Fujimoto reported the first case of KFD in 1972 and described it as lymphadenitis with reticular cell proliferation and abundant phagocytes and histocytes, suggesting benign histiocytic necrotizing lymphadenitis [6]. Although idiopathic with unclear etiology, several observations have postulated a viral origin to the disease. Viruses such as Ebstein-Barr virus (EBV), human herpesvirus (HHV) serotypes 6 and 8 have been causal factors [5,7]. An autoimmune etiology has also been implicated, as KFD has also been observed in systemic lupus erythematosus (SLE) [8]. It tends to have a geographic preference to the Asian population, which may be related to the genetic predisposition to the disease in people with human leukocyte antigen (HLA) class II alleles, HLA DPB1, and HLA DPA1, respectively, found in the Asian population and is uncommon in Caucasians [8].

Most common KFD manifestations include fever, weight loss, sweats, lymphadenopathy, anorexia, hepatomegaly, and lymphopenia. Several differential diagnoses are associated with this presentation [9]. The clinical presentation with a prodrome of sinopulmonary infection, lymphadenopathy, atypical lymphocytosis, and lack of antibiotic response strongly suggest the postulated viral etiology, even though the etiology remains largely unclear. The electron microscopic features suggestive of reticular tubular structures inside the cytoplasm of activated histiocytes and lymphocytes, which are found in KFD, have also been observed in other autoimmune disorders such as SLE, thus pointing to an autoimmune process as the possible etiology as well [1,10]. KFD is considered a self-limited immune-mediated disease induced by transformed lymphocytes infected with the virus. In addition, proliferating cytotoxic CD8+ lymphocytes initiating apoptosis may also be involved in disease pathogenesis [9,10].

Lymph node biopsy and immunohistochemistry are required for a definite diagnosis of KFD. The characteristic features on biopsy reveal asymmetrical paracortical areas of coagulative necrosis with karyorrhectic debris distorting the nodal architecture and a large number of myeloperoxidases, CD68+, CD128+, plasmacytoid dendritic cells, or activated CD8+ T lymphocytes [11]. The absence of neutrophils, granulomatous infection, incomplete effacement of the nodal architecture with patent sinuses, numerous atypical reactive histiocytes with relatively low mitotic rates, and absence of Reed-Sternberg (RD) cells differentiate KFD from SLE, tuberculosis (TB), and Hodgkin’s lymphoma, respectively [5,11]. Due to the striking similarity in the bundle of non-specific signs and symptoms overlapping with several conditions, a thorough workup and detailed investigations are mandated to differentiate the above conditions from KFD [12].

KFD management involves supportive as well as a specific treatment. In KFD, spontaneous resolution is typical, and treatment is warranted in certain conditions. Symptomatic treatment is the most effective treatment strategy for KFD. Supportive measures include non-steroidal anti-inflammatory drugs and antipyretics to relieve fever, lymph nodal tenderness, malaise, and arthralgias. Corticosteroids are reserved for severe cases or where supportive measures fail to control symptoms [13]. Overall, the disease course is benign with spontaneous self-resolution within weeks to months and a 3-4% low recurrence rate [14]. However, long-term follow-up of KFD is recommended, especially for its recurrence due to its postulated association as a precursor to autoimmune diseases like SLE.

## Conclusions

Although a rare disease, KFD should be kept in mind in the differential diagnosis of cervical lymphadenopathy. Due to the striking similarity in the bundle of non-specific signs and symptoms and presentations overlapping with several conditions such as TB, SLE, and lymphoma, a thorough workup and intense investigations are mandated to differentiate the above conditions from KFD. The diagnosis is confirmed by lymph node biopsy and immunohistochemistry. A long-term follow-up is required in patients with KFD to ascertain resolution and detection of recurrence.
